# Soybean meal mitigates vasoconstriction and serotonin suppression during fescue toxicosis in beef cattle

**DOI:** 10.1093/jas/skag108

**Published:** 2026-04-10

**Authors:** Luiz C O Sousa, Brittany E Davis, David L Harmon, Ronald J Trotta

**Affiliations:** Department of Animal and Food Sciences, University of Kentucky, Lexington, KY 40546, United States; Department of Animal Science, Universidade Federal de Viçosa, Viçosa, MG 36570-900, Brazil; Forage-Animal Production Research Unit, USDA-ARS, Lexington, KY 40546, United States; Department of Animal and Food Sciences, University of Kentucky, Lexington, KY 40546, United States; Department of Animal and Food Sciences, University of Kentucky, Lexington, KY 40546, United States

**Keywords:** blood flow, ergot alkaloid, fescue toxicosis, ruminant, serotonin, soybean

## Abstract

Legume supplementation has been shown to mitigate fescue toxicosis in beef cattle. However, no studies have directly compared the use of soybean-based feeds to alleviate symptoms of fescue toxicosis under controlled environmental heat and equalized feed intake. The objectives were to evaluate the effects of soybean hulls, whole soybeans, or soybean meal (SBM) supplementation on the mitigation of vasoconstriction, heat stress, and inflammatory responses in beef cattle during fescue toxicosis. Ten ruminally-cannulated Angus × Holstein steers were used in a 5 × 10 Latin rectangle design. Five treatments were evaluated: 1) non-toxic endophyte-infected tall fescue seed (NTE), 2) toxic endophyte-infected tall fescue seed (TE), 3) TE + soybean hulls (SBH), 4) TE + whole soybean (WSB), or 5) TE + SBM. Treatment periods lasted 7 d, followed by a 14-d washout. Heat stress was induced by maintaining room temperature at 32 °C during the light phase and 21 °C during the dark phase. Feed intake and feeding behavior were recorded daily. Caudal artery cross-sectional area (CAA) and hemodynamics were assessed before feeding (0 h), 4 h, and 8 h after the morning feeding on days −1 (baseline) and 7. Skin and rectal temperatures, respiration rate, blood pressure, heart rate were collected at 0 h, 4 h, and 8 h on days 1 and 7. Blood was collected at 0 h and 4-h on days 1 and 7. Treatments did not influence (*P *> 0.84) total dry matter (DM) intake. Steers receiving TE or TE + WSB consumed a greater (*P *= 0.02) number of meals compared with NTE and TE + SBH steers. Relative CAA decreased (*P *= 0.02) by 15.1 percentage units for TE compared with NTE and increased by 19 percentage units for TE + SBM compared with TE. Systolic blood pressure tended to increase (*P *= 0.06) in steers fed TE compared with steers receiving NTE or TE + SBM. Respiration rate, rectal temperature, and neck skin temperature were not influenced by soybean feed supplementation (*P *≥ 0.24). Rump skin temperature decreased (*P *= 0.02) in steers receiving TE compared with NTE and TE + SBM. SBM supplementation increased (*P *= 0.01) serum serotonin concentration by up to 34% compared with other TE-containing treatments. Dietary supplementation of soybean-based feeds has the potential to alleviate symptoms of fescue toxicosis in cattle. Among soybean-based feeds evaluated, SBM was the most effective because of the mitigation of ergot alkaloid-induced vasoconstriction, promotion of vasodilation, and restoration of serum serotonin and normal feeding behavior in beef cattle challenged with fescue toxicosis.

## Teaser

Soybean meal supplementation mitigated ergot alkaloid-induced vasoconstriction, promoted peripheral vasodilation, and restored serum serotonin and normal feeding behavior in beef cattle challenged with fescue toxicosis.

## Introduction

Most tall fescue (*Lolium arundinaceum*) is infected with the endophyte, *Epichlöe coenophialia*, that enhances strand persistence, forage productivity, and drought tolerance (Dinkins et al. 2017). However, the endophyte produces ergot alkaloids that impose toxic effects in ruminant livestock, resulting in fescue toxicosis, resulting in negative impacts on animal growth, reproduction, and lactation (Strickland et al. 2011; Klotz 2015). Symptoms of fescue toxicosis include decreases in feed intake, body weight gain, milk production, and reproductive performance and increases in respiration rate, body temperature, and vasoconstriction (Strickland et al. 2011). Mechanisms describing how ergot alkaloid ingestion relates to the development of clinical symptoms of fescue toxicosis are not completely understood. Ergot alkaloids share structural similarity with monoamine neurotransmitters, such as serotonin, dopamine, epinephrine, and norepinephrine (Berde 1980), enabling interactions with their respective receptors to alter numerous physiological and metabolic functions (Klotz 2015). It has been estimated that fescue toxicosis decreases the weaning weight of over 9 million beef calves in the Southeastern United States every year (Kallenbach 2015), translating to a $2 billion annual loss in economic revenue for the beef industry (Kallenbach 2015), and these losses have been estimated to increase over the last 10 yr. There are few solutions that are both economically feasible and effective in preventing the onset or promoting amelioration of fescue toxicosis.

Some studies have reported positive effects when supplementing soy-based feeds to ruminants grazing toxic tall fescue or exposed to ergot alkaloids because of greater concentrations of plant secondary compounds or increased nutritive value. Soybean hull supplementation increased average daily gain, increased serum prolactin, and improved hair coat scores of steers grazing toxic endophyte-infected tall fescue (Aiken et al. 2008; Carter et al. 2010). Soybean meal supplementation partially attenuated ergot alkaloid-induced vasoconstriction of the carotid artery in goats (Harlow et al. 2022). However, interpretation of these responses is complicated by potential confounding effects from decreased dry matter (DM) intake associated with ergot alkaloid ingestion and increased DM intake with supplemental feeding. Differences in nutrient intake, dilution of ergot alkaloid intake, and uncontrolled environmental conditions have made it difficult to delineate factors associated with the onset and amelioration of fescue toxicosis in grazing ruminants (Koontz et al. 2012; Nicol and Klotz 2015).

To our knowledge, no studies have directly compared the effects of soybean hull, whole soybean, or soybean meal supplementation on physiological responses to a fescue toxicosis challenge in beef cattle with controlled environmental heat loads and equalized feed intakes. Therefore, the objectives of this experiment were to evaluate the effects of soybean hulls, whole soybeans, and soybean meal supplementation on the mitigation of fescue toxicosis in beef cattle. The hypothesis of this experiment is soybean hull, whole soybean, and soybean meal supplementation will mitigate clinical symptoms of fescue toxicosis, including peripheral vasoconstriction, serotonin suppression, increased susceptibility to heat stress, and increased immune system activation.

## Materials and methods

All animal care and handling procedures were approved by the University of Kentucky Animal Care and Use Committee (2023-4296).

### Animals, diet, and housing

Ten Angus × Holstein steers (body weight [BW] = 221 ± 5.3 kg) were ruminally cannulated (Harmon and Richards 1997) for use in a 5 × 10 Latin Rectangle design experiment. Steers were housed indoors in individual pens (3 m × 3 m) in the Intensive Research Building of the University of Kentucky C. Oran Little Research Center in Versailles, KY. To simulate summer environmental conditions, the room was maintained with a 16:8 h light: dark cycle, and ambient temperature was increased to 32 °C during the light phase and decreased to 21 °C during the dark phase (Koontz et al. 2012; Valente et al. 2020). Steers were initially fed a corn silage-based diet devoid of soy-based feed ingredients (Table 1) once daily to supply 1.5 times the net energy required for maintenance (NASEM 2016).

**Table 1:** Composition of basal diet fed to steers.

Item	
**Ingredient composition**	
** Corn silage, %**	72.5
** Dried corn distillers’ grains with solubles, %**	17.5
** Finely ground corn, %**	7.18
** Limestone, %**	1.76
** Trace mineral premix, %^1^**	0.46
** Urea, %**	0.33
** Tallow, %**	0.23
** Vitamin A, D, & E premix, %^2^**	0.02
** Rumensin 90, %^3^**	0.01
** Tylan 40, %^4^**	0.01
**Chemical composition**	
** Dry matter, %**	40.4
** Total starch, % of DM**	40.1
** Neutral detergent fiber, % of DM**	27.7
** Acid detergent fiber, % of DM**	16.2
** Crude protein, % of DM**	10.3
** Crude fat, % of DM**	4.17
** Ca, % of DM**	0.780
** P, % of DM**	0.290
** Net energy for maintenance, Mcal/kg^5^**	1.72
** Net energy for gain, Mcal/kg^5^**	1.10

1 Contained: 56.34% Cl, 36.53% Na, 1.2% S, 0.06% Ca, 9.29 g Fe/kg, 5.52 g Zn/kg, 4.79 g Mn/kg, 1.84 g Cu/kg, 120 mg I/kg, 68.9 mg Co/kg, and 18.5 mg Se/kg on a DM basis.

2 Composed of vitamin A acetate (1,814 kIU/kg), D-activated animal sterol (source of vitamin D_3_; 363 kIU/kg), vitamin E supplement (227 IU/kg), roughage products, calcium carbonate, and mineral oil.

3 Contained 199 mg monensin per gram of premix.

4 Contained 18.1 g tylosin phosphate per kilogram of premix.

5 Calculated according to NASEM (2016).

### Experimental design and treatments

Rows and columns of the Latin Rectangle were randomized, and steers were randomly assigned to treatment sequences. During each experimental period, two steers received one of five treatments: 1) non-toxic novel endophyte-infected tall fescue seed (NTE; Estancia Forage Tall Fescue Grass Seed; 0 mg ergovaline + ergovalinine/kg; negative control), 2) toxic endophyte-infected tall fescue seed (TE; Kentucky 31 Tall Fescue Grass Seed; 9.34 mg ergovaline + ergovalinine/kg; positive control), 3) TE supplemented with soybean hulls (TE + SBH), 4) TE supplemented with raw whole soybeans (TE + WSB), and 5) TE supplemented with solvent-extracted soybean meal (TE + SBM). All seeds and supplements were dried to equalize DM concentration and then ground through a 3-mm screen using a hammer mill to minimize variation in particle size. Ground seeds and supplemental treatments were administered through the rumen cannula immediately before feeding (Koontz et al. 2012; Ahn et al. 2020; Valente et al. 2020). The TE dose provided 15 µg ergovaline + ergovalinine⋅kg BW^−1^⋅d^−1^, which was previously shown to effectively induce fescue toxicosis and reduce serum serotonin concentration (Ahn et al. 2020; Valente et al. 2020). The amount of TE and NTE seeds administered were balanced to ensure equivalent daily seed intake. Soy-based feeds were supplemented at 2.75 g/kg of BW per day on a DM basis to provide approximately 680 g/d for a 250-kg steer. To equalize DM intake among treatments, steers receiving NTE and TE alone were supplemented with an equivalent amount of ground corn. The selected supplementation rate reflects common practices among beef producers feeding soy-based feeds to growing cattle (Lehmkuhler and VanValin 2021), allowed supplementation rates to be scaled with each experimental period, maintained dietary ether extract below 6% of DM, and was comparable to the 2.61 g/kg of BW dose used by Harlow et al. (2022), who reported increased carotid artery luminal diameter in goats ruminally dosed with TE seed and supplemented with soybean meal. Treatment periods were 7 d in duration and were separated by 14-d washout periods. During washout periods, steers were housed outdoors in group pens for the first 10 d and then returned indoors during the final 4 d to allow acclimation to housing conditions. Baseline measurements were collected on the final day of each washout period (d −1). During washout periods, steers were fed the basal corn-silage-based diet at 1.5 times the net energy required for maintenance.

### Dry matter intake, feeding behavior, and nutrient analysis

Dry matter intake was measured on an individual steer basis daily by weighing the feed offered and subtracting the orts for each steer. All steers were pair-fed to the treatment with the lowest feed intake based on the feed intake measured during the previous day (as fed). The basal diet and orts were collected daily and analyzed for DM concentration by forced-air oven drying at 105 °C for 24 h. Distribution of feed intake throughout the day was monitored at 1-min intervals using an electronic scale equipped with a data logger attached to each animal’s feed bunk (Campbell Scientific, Logan, Utah, USA). Feeding behavior variables, including meal frequency, meal size, and meal duration, were calculated for each animal on each experimental day (Egert-McLean et al. 2019).

Samples of the basal diet (Table 1) were collected for nutrient analysis, including DM, total starch, crude protein, crude fat, neutral detergent fiber, acid detergent fiber, and mineral concentrations. Diet samples were partially dried at 55 °C for 48 h in a forced-air oven and subsequently ground to pass a 1-mm screen using a Wiley mill. Dry matter concentration was determined by oven-drying for 3 h at 105 °C (NFTA 2.1.4.). Total starch concentration was determined according to AOAC method 2014.10 by first gelatinizing samples in water, followed by two-step enzymatic hydrolysis with α-amylase and glucoamylase in acetate buffer (AOAC 2023). Free glucose concentration was measured using the glucose oxidase electrode of a YSI Series 2950 D-1 Biochemistry Analyzer (YSI Inc., Yellow Springs, OH) and then multiplied by 0.9 to convert to anhydroglucose equivalents, as found in starch (McCleary et al. 1994). Nitrogen concentration was determined by combustion (AOAC 1999) (method 990.03) using a CN628 Carbon/Nitrogen Determinator (Leco Corporation, St. Joseph, MI), and crude protein concentration was calculated by multiplying N concentration × 6.25. Crude fat concentration was determined by ether extraction using an ANKOM X15 Extractor (ANKOM Technology Method 2, Macedon, NY). Neutral detergent fiber and acid detergent fiber concentrations were determined sequentially using the filter bag technique (ANKOM Technology Methods 14 and 15, respectively) with an automated fiber analyzer (ANKOM DELTA; ANKOM Technology, Macedon, NY). For mineral analysis, samples were digested in 50-mL MARSXPress vessels (CEM Corporation, Matthews, NC) using a MARS 6 Microwave Digestion System (CEM Corporation, Matthews, NC), and calcium and phosphorus concentrations were determined using inductively coupled plasma-optical emission spectroscopy (iCAP PRO XP ICP-OES; Thermo Fisher Scientific Inc., Beverly, MA). Total aglycone and individual isoflavone concentrations (Table 2) were quantified using liquid chromatography-mass spectrometry (Davis et al. 2023). Tall fescue seed samples were analyzed for ergovaline + ergovalinine concentrations using high-performance liquid chromatography with fluorescence detection (Yates and Powell 1988; Klotz et al. 2018).

**Table 2:** Chemical composition, individual and total aglycone concentration of the basal diet, corn, and soybean feeds.

Item	Feed				
	Basal diet	Corn	Soybean hulls	Whole soybean	Soybean meal
**Dry matter, %**	—	89.2	91.3	93.8	91.4
**Total starch, % DM**	—	67.2	0.20	1.60	0.70
**Neutral detergent fiber, % DM**	—	10.7	64.0	21.8	9.40
**Acid detergent fiber, % DM**	—	3.20	47.5	17.5	5.30
**Crude protein, % DM**	—	9.30	12.1	33.1	50.0
**Crude fat, % DM**	—	4.36	2.55	16.8	4.53
**Ca, % DM**	—	0.01	0.60	0.31	0.35
**P, % DM**	—	0.31	0.12	0.52	0.65
**NEg, Mcal/kg^1^**	—	1.53	1.27	2.55	2.09
**NEm, Mcal/kg^2^**	—	2.21	0.70	1.81	1.42
**Isoflavones, µg/g DM**					
** Biochanin A**	<10	<10	<10	<10	<10
** Formononetin**	<10	<10	<10	<10	<10
** Genistein**	<10	<10	66.5	601	860
** Daidzein**	<10	<10	91.0	478	705
** Total aglycones**	<10	<10	157.5	1,079	1,565

1 NEg, net energy for gain.

2 NEm, net energy for maintenance.

### Caudal artery hemodynamics

Caudal artery cross-sectional area and hemodynamics at the 4th coccygeal vertebrae were assessed using color Doppler ultrasonography (Terason usmart 3300 NexGen ultrasound; Terason, Burlington, MA, USA). Measurements were collected at 0-h (before feeding), 4-h and 8-h after the morning feeding on days −1 (baseline, last day of the washout period prior to treatment administration) and 7 of each experimental period. For assessment of caudal artery cross-sectional area, the uSmart 15L4 linear array transducer (12 MHz) was positioned at the 4th coccygeal vertebrae in cross-sectional orientation, and arterial pulsations were recorded as video clips (5 cm depth, 4.5 MHz frequency, 3.0 Hz pulse repetitive frequency, and 40.0 gain). The caudal artery diameter was traced during five separate peak systolic phases and averaged. Cross-sectional area was calculated as *π r^2^*, where *r* is the vessel radius (Aiken et al. 2007; Aiken et al. 2009; Davis et al. 2023).

Following the measurement of arterial cross-sectional area, the transducer was repositioned at the same anatomical location in longitudinal orientation, and three consecutive cardiac cycle waveforms were recorded (2-cm depth, 24˚ correction angle, 0.5 mm sample volume). Peak systolic velocity, end diastolic velocity, and heart rate were calculated using built-in software functions of the ultrasound instrument. Mean velocity was calculated manually by tracing each cardiac cycle waveform using the ultrasound software. Pulsatility index was calculated as the difference between peak systolic velocity and end diastolic velocity divided by mean velocity. Resistance index was calculated as the difference between peak systolic velocity and end diastolic velocity divided by peak systolic velocity. Blood flow was calculated as mean velocity × cross-sectional area × 60 s (Lemley et al. 2012). Stroke volume was calculated as blood flow (mL/min)/heart rate (beats/min). Doppler measurements obtained on day −1 were averaged across time and defined as baseline. Caudal artery cross-sectional area, blood flow, and stroke volume were expressed as absolute measurements and measurements relative to baseline. Measurements related to baseline were calculated as:


$$\text{Change}\;\text{from}\;\text{baseline}\;(\%)\;=\frac{{\mathrm{Response}}_{\mathrm{treatment}\;\mathrm{period}}-{\mathrm{Baseline}}_{\mathrm{washout}\;\mathrm{period}}\;}{{\mathrm{Baseline}}_{\mathrm{washout}\;\mathrm{period}}}\times 100$$

For measurements expressed relative to baseline, positive percent changes indicated vasodilatory activity and negative percent changes indicated vasoconstrictive activity.

### Physiological stress measures

Skin temperature (neck and rump), rectal temperature, respiration rate, heart rate, and blood pressure were assessed as indicators of heat stress and fescue toxicosis (Al-Qaisi et al. 2019). Measurements were recorded at 0 h (before feeding), 4 h, and 8 h after the morning feeding on days 1 and 7 of each experimental period. To facilitate accurate measurements, hair in the neck and rump areas were clipped on day −1 of each period, and skin temperatures were measured using a digital infrared thermometer (Fluke Corporation, Everett, WA, USA). Rectal temperature was measured using a digital thermometer (AG-Medix, Mukwonago, WI, USA). Respiration rate was determined by counting flank movements for 15 s and multiplying by four to obtain breaths per minute. Blood pressure and heart rate were measured using an adjustable (16 cm to 24 cm) blood pressure cuff that was wrapped around the tailhead and connected to a digital monitor (Lifesource A&D Engineering, Inc., San Jose, CA, USA) as described previously (Eisemann et al. 2014).

### Blood collection and analysis

Blood (40 mL) was collected from the jugular vein with a syringe on day 1 at 0 h (before feeding and treatment administration) and on day 7 at 4 h after feeding. Blood was dispensed from syringes into two 10-mL tubes containing K_2_EDTA and two 10-mL tubes with clot activator. Steers had continuous access to feed and water during blood sampling. Vacutainer tubes for plasma were centrifuged immediately after collection, whereas vacutainer tubes for serum were allowed to clot at room temperature for 45 min to 60 min before centrifugation. Whole blood was centrifuged (2,000 × *g*; 20 min; 4 °C) and the supernatants were aliquoted into 2-mL tubes and stored at −80 °C until analysis.

Plasma glucose concentration was analyzed using the hexokinase/glucose-6-phosphate dehydrogenase method (Farrance 1987) with Infinity Hexokinase Reagent (ThermoFisher Scientific Inc., Middletown, VA, USA). Plasma non-esterified fatty acid (NEFA) concentration was analyzed using the acyl CoA synthetase-acyl CoA oxidase-peroxidase method (Okabe et al. 1980) with the LabAssay NEFA FFA kit (FUJIFILM Wako Pure Chemical Corporation, Richmond, VA, USA). Plasma β-hydroxybutyrate concentration was analyzed using the β-hydroxybutyrate dehydrogenase/tetrazolium salt method (McMurray et al. 1984) with the Fisherbrand β-Hydroxybutyrate Liquicolor Reagent Kit (Stanbio Laboratory L.P., Boerne, TX, USA). Plasma urea concentration was quantified using the *o*-phthaldialdehyde/primaquine diphosphate method described by Jung et al. (1975) and modified by Zawada et al. (2009). All colorimetric assays were adapted for use with a multi-mode plate reader (BioTek Synergy HTX; Agilent Technologies Inc., Santa Clara, CA, USA). Detailed protocols are provided in Supplementary Materials and Methods.

Acute-phase proteins (serum amyloid A, haptoglobin) have been used as positive biomarkers of immune system activation and inflammation in cattle (Kvidera et al. 2017; Horst et al. 2020; Pate et al. 2021). Cholesterol, total bilirubin, and albumin have been used as negative biomarkers of immune system activation and inflammation in cattle (Rodriguez-Jimenez et al. 2025). Serum amyloid A (TP807; Tridelta Phase range SAA, Tridelta Development Ltd., Maynooth, Co. Kildare, Ireland) and haptoglobin (HAPT-11; Cow Haptoglobin ELISA Kit; Life Diagnostics, West Chester, PA, USA) concentrations were analyzed using ELISA kits according to the manufacturer’s instructions. Plasma albumin concentration was quantified using the bromocresol green method (Doumas et al. 1971). Plasma cholesterol concentration was analyzed using the cholesterol esterase-cholesterol oxidase-peroxidase method (Allain et al. 1974; Roeschlau et al. 1974) with Infinity Cholesterol Liquid Stable Reagent (Thermo Fisher Scientific Inc., Waltham, MA, USA). Serum total bilirubin concentration was analyzed using the Jendrassik-Grof method (Shull et al. 1980). All procedures were adapted for use with a multi-mode plate reader (BioTek Synergy HTX; Agilent Technologies Inc., Santa Clara, CA, USA). Full procedure descriptions are available in Supplementary Materials and Methods.

The concentrations of tryptophan, kynurenine, 5-hydroxytryptophan (5-HTP), serotonin, and 5-hydroxyindolacetic acid (5-HIAA) in serum were quantified by high-performance liquid chromatography (HPLC) with modified methods from Sa et al. (2012). Briefly, 500 µL of serum was deproteinized by mixing with an equal volume of 5% (v/v) perchloric acid containing 50 mM kynurenic acid for use as an internal standard. Samples were chilled at 4 °C for 5 min, vortexed, and centrifuged (20,000 × g; 10 min; 4 °C). The acidified supernatants (800 µL) were transferred to 2 mL new tubes and buffered with 410 µL of 0.6 M KOH. The samples were chilled at 4 °C for 5 min and centrifuged (20,000 × g; 10 min; 4 °C). A 10-µL aliquot of the resulting supernatant was then injected into the HPLC system (1260 Infinity II HPLC System; Agilent Technologies Inc., Santa Clara, CA, USA) using an autosampler. Chromatographic separation was performed using an ACE C18-PFP column (4.6 × 150 mm, 3 µm; Advanced Chromatography Technologies, Aberdeen, Scotland) maintained at 39 °C. The mobile phase consisted of 50 mM KH_2_PO_4_ and methanol (85:15 v/v; pH = 4.3) at a flow rate of 1.0 mL/min. Separated tryptophan metabolites were detected by fluorescence (tryptophan, 5-HTP, serotonin, 5-HIAA) or UV detection (kynurenine). The fluorescence detector was operated with an excitation wavelength of 278 nm and an emission wavelength of 338 nm, and the UV detector was set at 365 nm. The concentrations of tryptophan metabolites were quantified based on peak areas relative to a mixed analytical standard solution [50 µM L-tryptophan (T-0254; Sigma-Aldrich), 5 µM L-kynurenine (K9625; Sigma-Aldrich), 5 µM 5-hydroxy-L-tryptophan (H0531, TCI AMERICA), 5 µM serotonin hydrochloride (S0370, TCI AMERICA), and 5 µM 5-hydroxyindole-3-acetic acid (H8876; Sigma-Aldrich)] containing 15 µM kynurenic acid in KH_2_PO_4_ (pH = 4.3). Peak integration was performed electronically using OpenLAB ChemStation software (Agilent Technologies Inc., Santa Clara, CA, USA).

### Total-tract gastrointestinal permeability

Total-tract gastrointestinal permeability was assessed using Cr-EDTA as a paracellular leak permeability marker (Wood et al. 2015; Horst et al. 2020; Bertens et al. 2024). On d 4, temporary intravenous catheters (14 Ga × 13 cm, Mila International, Inc., Florence, KY, USA) were inserted into the jugular vein. Before feeding and treatment administration on day 5, Cr-EDTA solution (180 mM) (Binnerts et al. 1968) was pulse-dosed through the ruminal cannula at a dose of 0.369 mmol/kg BW (Bertens et al. 2024). Blood (20 mL) was collected through the jugular vein catheter at 2, 8, 20, 40, and 48 h after Cr-EDTA infusion (Bertens et al. 2024) and added to two 10-mL Vacutainer tubes containing sodium heparin. After collection of individual blood samples, 10 mL of heparinized saline was infused through the jugular catheter to flush residual blood and maintain patency.

Vacutainer tubes were centrifuged (2,500 × *g*; 15 min; 4 °C), plasma was aliquoted into 2-mL tubes, and stored at −80 °C. Plasma Cr concentrations were determined using atomic absorption spectroscopy (AAnalyst 200; PerkinElmer Inc., Shelton, CT, USA). Absorbance was measured at 357.87 nm. Plasma Cr area under the curve was calculated using the trapezoidal method in GraphPad Prism 9.4 (GraphPad Software, San Diego, CA, USA).

### Statistical analyses

All analyses were performed using the GLIMMIX procedure of SAS (version 9.4; SAS Institute Inc., Cary, NC). For variables containing repeated measures, the fit of covariance structures was evaluated and selected based on the smallest Akaike’s information criterion with correction (AICC). Feed intake and feeding behavior variables were analyzed as repeated measures using the random statement. The model included fixed effects of treatment, day, and the day × treatment interaction with random effects of animal and period. Caudal artery hemodynamics and plasma Cr concentrations were analyzed using the repeated measures statement, including the fixed effects of treatment, time, and the time × treatment interaction with random effects of animal and period. Baseline measurements of caudal artery hemodynamic variables were included as covariates in the model. Plasma and serum metabolites, physiological stress indicators, and plasma Cr AUC were analyzed considering the fixed effect of treatment and the random effects of animal and period. Outliers were identified and removed if studentized residuals exceeded ± 2.5 (single measures) or ± 3.0 (repeated measures). Degrees of freedom were estimated using the Kenward–Roger method. Least squares means were compared using Fisher’s least significant difference approach, protected by a significant *F*-test. Statistical significance was declared at *P *< 0.05, and tendencies were declared when 0.05 < *P *< 0.10.

## Results

### Dry matter intake and feeding behavior

There were no interactions (*P *≥ 0.16) between treatments and days for feed intake and feeding behavior variables (Table 3). Treatments did not influence (*P *≥ 0.35) total DM intake, basal diet DM intake, meal duration, or meal size. Steers receiving TE or TE + WSB consumed a greater (*P *= 0.02) number of meals compared with NTE and TE + SBH steers.

**Table 3:** Effects of soybean hulls, whole soybeans, or soybean meal on feed intake and feeding behavior in steers receiving endophyte-infected tall fescue seed.

Item	Treatment^1^					SEM^2^	*P-*value^3^		
	NTE	TE	TE + SBH	TE + WSB	TE + SBM		Trt	Day	Day × Trt
**Total DM intake, kg**	4.47	4.34	4.33	4.32	4.38	0.322	0.84	<0.01	0.96
**Basal diet DM intake, kg**	3.37	3.26	3.24	3.23	3.31	0.269	0.80	<0.01	0.99
**Supplement DM intake, kg**	0.690	0.686	0.698	0.670	0.674	0.042	0.35	–	–
**Fescue seed DM intake, kg**	0.395	0.399	0.399	0.399	0.399	0.013	–	–	–
**Number of meals**	6.13^b^	7.24^a^	5.95^b^	7.28^a^	6.62^a,b^	0.670	0.02	<0.01	0.16
**Meal duration, min**	24.9	20.8	23.8	20.2	23.4	3.16	0.47	<0.01	0.18
**Meal size, kg as-fed**	1.28	1.03	1.36	1.02	1.16	0.183	0.45	<0.01	0.35

1 NTE, non-toxic endophyte; TE, toxic endophyte; TE + SBH, toxic endophyte + soybean hulls; TE + WSB, toxic endophyte + whole soybean; TE + SBM, toxic endophyte + soybean meal.

2 SEM, standard error of the mean (*n* = 10 steers per treatment).

3 Trt, main effect of treatment; Day, main effect of experimental day; Day × Trt: interaction of treatment with experimental day.

a,b Means within a row with different superscripts differ at *P *< 0.05.

### Caudal artery hemodynamics

There was a tendency for time × treatment interaction for heart rate (*P *= 0.06) and blood flow relative to baseline (*P *= 0.08; Table 4). Heart rate did not differ (*P *≥ 0.15) among treatments before feeding or 8 h after feeding. At 4 h after feeding, steers receiving TE + WSB and TE + SBM had greater heart rate (*P *≤ 0.05) compared with those receiving TE and TE + SBH, whereas the NTE showed an intermediate response. Treatments did not influence (*P *≥ 0.16) blood flow relative to baseline before feeding and 4 h after feeding. At 8 h after feeding, steers receiving TE had decreased (*P *< 0.05) blood flow by 24.2 percentage units compared with NTE. Compared with TE, supplementation with TE + SBH and TE + SBM increased (*P *< 0.05) blood flow by 23.4 and 19.8 percentage units, respectively. The time × treatment interactions were not significant (*P *≥ 0.39) for other hemodynamic variables (Table 4).

**Table 4:** Effects of soybean hulls, whole soybeans, or soybean meal on caudal artery hemodynamics in steers receiving endophyte-infected tall fescue seed.

Item	**Treatment** ^1^						***P*-value** ^3^		
	NTE	TE	TE + SBH	TE + WSB	TE + SBM	**SEM** ^2^	Trt	Time	Time × Trt
**Cross-sectional area, mm** ^2^	19.1^x,y^	17.0^z^	18.6^x,y^	18.1^y,z^	19.6^x^	0.73	0.06	<0.01	0.92
**Cross-sectional area, % change from baseline**	6.51^a,b^	−8.62^c^	2.42^a,b^	−0.378^b,c^	10.4^a^	4.67	0.02	<0.01	0.70
**Peak systolic velocity, cm/s**	22.3	22.9	23.8	22.9	22.9	1.08	0.84	0.02	0.43
**End diastolic velocity, cm/s**	2.68	2.81	2.75	2.84	2.81	0.085	0.59	<0.01	0.44
**Mean velocity, cm/s**	10.2	10.2	10.8	10.6	10.6	0.47	0.77	<0.01	0.39
**Resistance index**	0.874	0.873	0.876	0.869	0.877	0.007	0.84	0.08	0.78
**Pulsatility index**	1.93^a,b^	1.96^a^	1.97^a^	1.85^c^	1.90^b,c^	0.026	0.01	<0.01	0.66
**Heart rate**	70.2^b^	71.8^b^	70.6^b^	77.9^a^	73.8^a,b^	3.06	0.02	<0.01	0.06
**Blood flow, mL/min**	121	106	121	113	123	8.05	0.23	<0.01	0.47
**Blood flow, % change from baseline**	1.73^x,y,z^	−7.91^z^	4.38^x,y^	−1.38^y,z^	12.9^x^	6.69	0.08	<0.01	0.08
**Stroke volume, mL/beat**	1.71	1.51	1.72	1.51	1.66	0.161	0.12	<0.01	0.38
**Stroke volume, % change from baseline**	0.145	0.025	0.135	0.036	0.178	0.101	0.18	0.015	0.32

1 NTE, non-toxic endophyte; TE, toxic endophyte; TE + SBH, toxic endophyte + soybean hulls; TE + WSB, toxic endophyte + whole soybean; TE + SBM, toxic endophyte + soybean meal.

2 SEM, standard error of the mean (*n* = 10 steers per treatment).

3 Trt, main effect of treatment; Time, main effect of time; Time × Trt: interaction of time with treatment.

a,b,c Means within a row with different superscripts differ at *P *< 0.05.

x,y,z Means within a row with different superscripts differ at *P *< 0.10.

Caudal artery cross-sectional area tended to decrease (*P *= 0.06) for steers ruminally dosed with TE (Table 4). When expressed relative to baseline, caudal artery cross-sectional area decreased (*P *= 0.02) by 15.1 percentage units for steers receiving TE compared with NTE. The relative caudal artery cross-sectional area increased (*P *= 0.02) by 19 and 11 percentage units for steers ruminally dosed with TE + SBM or TE + SBH compared with TE, respectively. Relative caudal artery cross-sectional area did not differ (*P *≥ 0.15) for TE + WSB compared with NTE or TE controls. Peak systolic velocity, end diastolic velocity, mean velocity, and resistance index were not influenced (*P *≥ 0.59) by soybean feed supplementation. Steers receiving TE and TE + SBH had greater (*P *= 0.01) pulsatility indexes compared with steers receiving TE + WSB. Absolute blood flow and stroke volume were not influenced (*P *≥ 0.12) by soybean feed supplementation.

### Physiological stress indicators

Treatments did not influence (*P *≥ 0.38) rectal temperature or neck temperature (Table 5). Conversely, rump skin temperature decreased (*P *= 0.02) in steers receiving TE compared with NTE and TE + SBM. Skin temperatures were intermediate in steers fed TE + SBH and TE + WSB, not differing from the other treatments (*P *> 0.05). Respiration rate was not affected by treatments (*P *= 0.24). Treatments tended to affect systolic pressure (*P *= 0.06) but did not influence diastolic pressure (*P *= 0.67). Systolic pressure tended to increase (*P *= 0.06) in TE steers compared with NTE steers. Systolic pressure tended to decrease (*P *= 0.06) in TE + SBM steers compared with TE steers. Furthermore, steers receiving NTE, TE + SBM, and TE + WSB showed greater (*P *< 0.01) heart rate compared with those fed TE and TE + SBH.

**Table 5:** Effects of soybean hulls, whole soybeans, or soybean meal on physiological stress indicators in steers receiving endophyte-infected tall fescue seed.

Item	**Treatment** ^1^					**SEM** ^2^	*P*-value
	NTE	TE	TE + SBH	TE + WSB	TE + SBM		
**Rectal temperature, °C**	39.4	39.2	39.3	39.3	39.2	0.149	0.38
**Neck temperature, °C**	37.2	36.9	37.0	37.0	37.1	0.226	0.64
**Rump temperature, °C**	37.0^a^	36.6^b^	36.9^a,b^	36.8^a,b^	37.1^a^	0.217	0.02
**Respiration rate, breaths/min**	72.4	78.8	74.4	85.1	84.2	7.89	0.24
**Systolic pressure, mm Hg**	95.6^y,z^	104^x^	102^x,y^	98.6^x,y,z^	93.0^z^	4.42	0.06
**Diastolic pressure, mm Hg**	46.6	48.5	46.2	46.1	45.8	2.87	0.67
**Heart rate, beats/min**	87.2^a^	82.4^b^	80.0^b^	90.6^a^	91.3^a^	3.95	<0.01

1 NTE, non-toxic endophyte; TE, toxic endophyte; TE + SBH, toxic endophyte + soybean hulls; TE + WSB, toxic endophyte + whole soybean; TE + SBM, toxic endophyte + soybean meal.

2 SEM, standard error of the mean (*n* = 10 steers per treatment).

a,b Means within a row with different superscripts differ at *P *< 0.05.

x,y,z Means within a row with different superscripts differ at *P *< 0.10.

### Serotonin metabolites

Serum tryptophan concentration was not affected by treatments (*P *= 0.12; Table 6). Serum kynurenine concentration was affected by treatment (*P *= 0.03), as concentrations were greater for steers receiving TE + SBM and TE + SBH compared with NTE and TE controls. Serum 5-HTP concentration tended to be greater (*P *= 0.10) for all TE-containing treatments. Serum serotonin concentrations did not differ (*P *> 0.05) between NTE and TE. In contrast, steers receiving TE + SBM had greater (*P *≤ 0.05) serum serotonin concentrations than those fed TE, TE + SBH, or TE + WSB but did not differ from NTE (*P *> 0.05). Serum 5-HIAA concentration tended to be greater (*P *= 0.09) in steers fed all TE-containing treatments.

**Table 6:** Effects of soybean hulls, whole soybeans, or soybean meal on serum concentrations of tryptophan metabolites in steers receiving endophyte-infected tall fescue seed.

Item	**Treatment** ^1^					**SEM** ^2^	*P*-value
	NTE	TE	TE + SBH	TE + WSB	TE + SBM		
**Tryptophan, µM**	19.4	21.0	21.8	23.3	24.5	2.51	0.28
**Kynurenine, µM**	5.81^c^	5.81^c^	6.78^a,b^	6.32^b,c^	7.24^a^	0.54	0.03
**5-hydroxytryptophan, µM**	0.133^y^	0.163^x^	0.159^x^	0.155^x^	0.155^x^	0.014	0.10
**Serotonin, µM**	10.2^a,b^	9.78^b^	8.33^c^	9.84^b^	11.2^a^	1.03	0.01
**5-hydroxyindoleacetic acid, µM**	0.367^y^	0.409^x^	0.393^x^	0.398^x^	0.390^x,y^	0.029	0.09

1 NTE, non-toxic endophyte; TE, toxic endophyte; TE + SBH, toxic endophyte + soybean hulls; TE + WSB, toxic endophyte + whole soybean; TE + SBM, toxic endophyte + soybean meal.

2 SEM, standard error of the mean (*n* = 10 steers per treatment).

a,b,c Means within a row with different superscripts differ at *P *< 0.05.

x,y Means within a row with different superscripts differ at *P *< 0.10.

### Metabolic and inflammatory biomarkers

Plasma glucose concentration was greater (*P *= 0.02) in all TE-containing treatments compared with those receiving NTE (Table 7). Plasma NEFA concentration was not affected by treatments (*P *= 0.25). Conversely, plasma β-hydroxybutyrate concentration was greater (*P *= 0.02) in steers fed NTE compared with those receiving TE. Plasma urea concentration was greater (*P *< 0.01) in steers receiving NTE compared with those receiving TE. Among soybean-fed steers, plasma urea concentration was greatest in TE + SBM, intermediate in TE + WSB, and least in TE + SBH (*P *< 0.01).

**Table 7:** Effects of soybean hulls, whole soybeans, or soybean meal on metabolic and inflammatory biomarkers in steers receiving endophyte-infected tall fescue seed.

Item	**Treatment** ^1^					**SEM** ^2^	*P*-value
	NTE	TE	TE + SBH	TE + WSB	TE + SBM		
**Metabolic markers**							
** Glucose, mM**	3.73^b^	4.13^a^	4.09^a^	4.23^a^	4.29^a^	0.201	0.02
** Non-esterified fatty acids, mM**	99.8	101	111	125	96.4	16.5	0.25
** β-hydroxybutyrate, mM**	0.881^a^	0.627^b^	0.706^b^	0.704^b^	0.712^b^	0.058	0.02
** Urea, mM**	1.40^d^	1.04^e^	1.70^c^	3.00^b^	3.29^a^	0.21	<0.01
**Positive inflammatory markers**							
** Amyloid A, µg/mL**	69.2^y,z^	57.3^z^	83.4^x,y^	82.4^x,y,z^	98.2^x^	32.2	0.08
** Haptoglobin, µg/mL**	104	194	155	74.1	87.2	93.0	0.31
**Negative inflammatory markers**							
** Albumin, g/dL**	2.85	2.80	2.83	2.82	2.87	0.075	0.88
** Bilirubin, mg/dL**	0.725^b,c^	0.797^a,b^	0.859^a^	0.776^a,b^	0.675^c^	0.059	0.03
** Cholesterol, mg/dL**	68.9^b,c^	68.3^c^	85.3^a^	75.7^a,b^	70.7^b,c^	4.77	0.01

1 NTE, non-toxic endophyte; TE, toxic endophyte; TE + SBH, toxic endophyte + soybean hulls; TE + WSB, toxic endophyte + whole soybean; TE + SBM, toxic endophyte + soybean meal.

2 SEM, standard error of the mean (*n* = 10 steers per treatment).

a,b,c Means within a row with different superscripts differ at *P *< 0.05.

x,y,z Means within a row with different superscripts differ at *P *< 0.10.

Serum amyloid A concentration tended to differ among treatments (*P *= 0.08), as concentrations were generally greater in the soy-containing treatments. Serum haptoglobin and plasma albumin concentrations were not affected by treatments (*P *> 0.30). Serum total bilirubin concentration was increased (*P *= 0.03) for the TE-containing treatments with the exception of TE + SBM, which was lower and similar to NTE. There were no differences between NTE, TE, and TE + SBM on plasma cholesterol concentration (*P *> 0.05). However, plasma cholesterol concentration was greater (*P *= 0.01) in steers receiving TE + SBH and intermediate for steers fed TE + WSB.

### Total-tract gastrointestinal permeability

**Figure 1:**
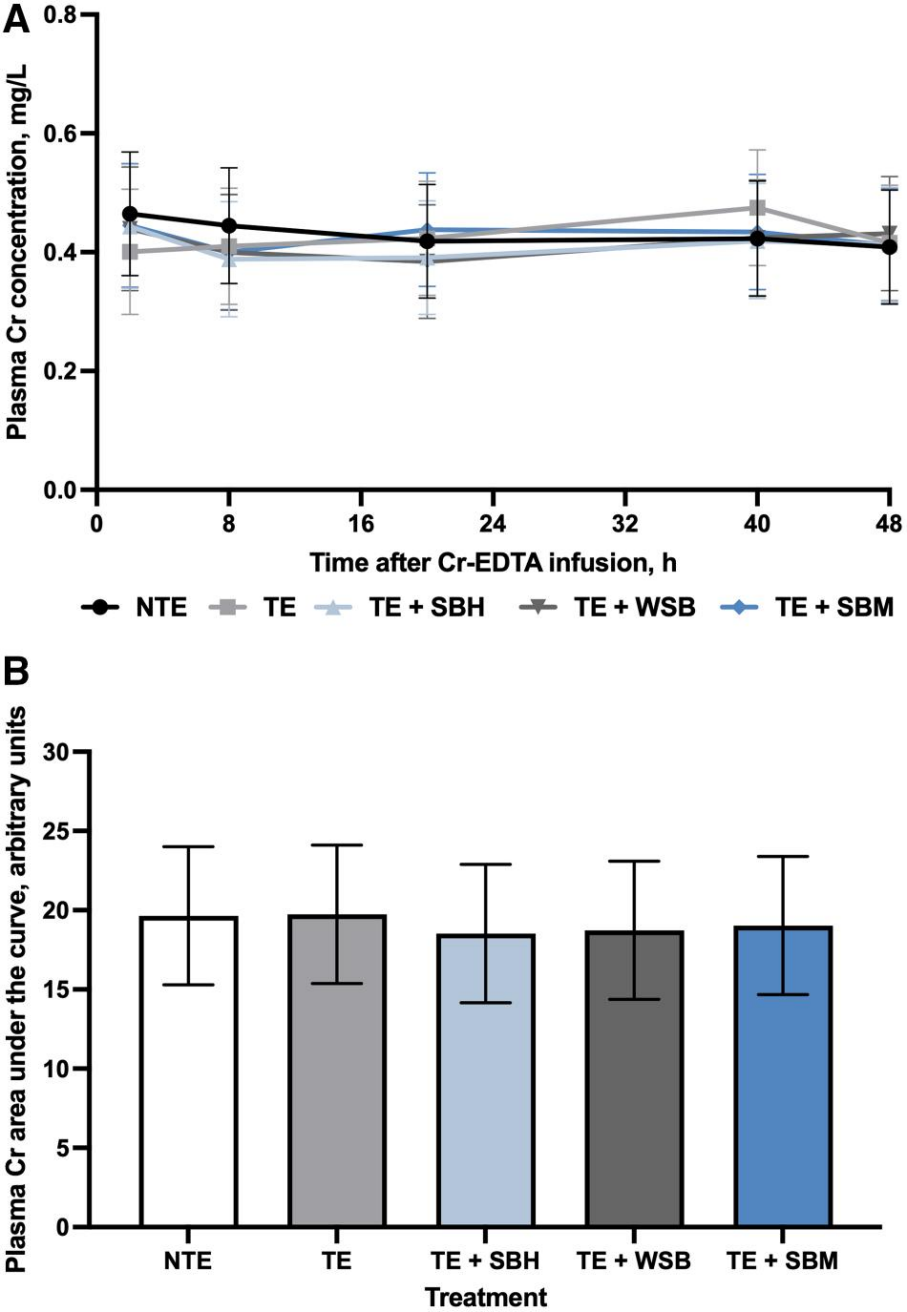
Influence of soybean hull, whole soybean, or soybean meal supplementation to steers receiving toxic endophyte-infected tall fescue seed on total-tract gastrointestinal permeability. A) Plasma Cr concentration at 2, 8, 20, 40, and 48 h relative to ruminal CrEDTA dosing. Probability values for time: *P *= 0.03, treatment: *P *= 0.72, and the time × treatment interaction: *P *= 0.77. *n* = 10 steers per treatment. B) Plasma Cr area under the curve of steers receiving endophyte-infected tall fescue seed. Probability value for treatment: *P *= 0.16. *n* = 10 steers per treatment. NTE, non-toxic endophyte-infected tall fescue; TE, toxic endophyte-infected tall fescue; TE + SBH, toxic endophyte-infected tall fescue + soybean hulls; TE + WSB, toxic endophyte-infected tall fescue + whole soybean; TE + SBM, toxic endophyte-infected tall fescue + soybean meal.

There was no effect of treatments (*P *= 0.72) or interaction between treatments and time (*P *= 0.77) on plasma Cr concentrations (Figure 1A). Additionally, the plasma Cr AUC was not affected by treatments (*P *= 0.16; Figure 1B).

## Discussion

The objectives of the current study were to evaluate the effects of SBH, WSB, and SBM supplementation on ergot alkaloid-induced vasoconstriction, susceptibility to heat stress, and systemic immune activation in beef cattle during fescue toxicosis. Previous studies have reported benefits of soybean feed supplementation in steers grazing toxic endophyte-infected tall fescue, including increased average daily gain (Aiken et al. 2008), restored serum prolactin, and improved hair coat scores (Carter et al. 2010). More recently, SBM supplementation partially alleviated ergot alkaloid-induced vasoconstriction in goats (Harlow et al. 2022). However, a clear mechanism explaining the magnitude of responses observed across studies remains difficult to delineate, partly due to potential confounding effects caused by decreased feed intake in animals exposed to ergot alkaloids and the effects of supplements on forage and ergot alkaloid intake. To our knowledge, no studies have directly compared use of soybean feeds to mitigate symptoms of fescue toxicosis in beef cattle with controlled environmental heat loads and equalized feed intakes.

### Mitigation of ergot alkaloid-induced vasoconstriction

One of the predominant negative symptoms of fescue toxicosis is increased vasoconstriction, leading to decreased peripheral blood flow in several ruminant livestock species (Aiken et al. 2007, 2009; Harlow et al. 2022; Davis et al. 2023). In the present study, TE ingestion decreased the cross-sectional area of the caudal artery by 8.62% and tended to decrease peripheral blood flow by 7.91% relative to baseline. Supporting these observations, systolic blood pressure of the caudal artery was 8.8% greater in TE steers compared with NTE steers. These results confirm that ergot alkaloid-induced vasoconstriction occurred in TE steers.

Previous research demonstrated that TE + SBM supplementation to goats increased carotid arterial cross-sectional area by 30.7% compared with goats receiving TE alone (Harlow et al. 2022). However, goats receiving TE had decreased hay intake compared with goats receiving TE + SBM, which prevented assessing the direct impact of SBM on alleviating ergot alkaloid-induced vasoconstriction (Harlow et al. 2022). In the current study, SBM supplementation to steers increased the relative cross-sectional area of the caudal artery by 20.8% compared with TE under pair-fed conditions. Likewise, SBM increased caudal arterial blood flow by 12.9% compared with NTE and by 22.6% relative to TE. These observations demonstrate that SBM supplementation mitigated ergot alkaloid-induced vasoconstriction and promoted peripheral vasodilation to increase blood flow in steers, independent of changes in feed intake.

Previous research has demonstrated that SBH supplementation increased the average daily gain of steers grazing TE pastures (Aiken et al. 2008; Carter et al. 2010), increased digestible organic matter intake and total-tract organic matter digestibility (Fieser and Vanzant 2004), and increased duodenal microbial N flow and nitrogen retention (Fieser and Vanzant 2004; Richards et al. 2006). In the current study, supplementing TE + also mitigated ergot alkaloid-induced vasoconstriction (+13.4% relative to TE) and increased caudal artery blood flow (+12.1% relative TE), although to a lesser extent than SBM supplementation. It is possible that beneficial effects of SBH supplementation could result from positive associative effects on forage intake, digestibility, and nitrogen utilization, and/or increased estrogenic activity mediated by isoflavones (Fieser and Vanzant 2004; Richards et al. 2006; Shappell et al. 2015).

Legumes, including red clover, white clover, and soybean-based feeds, contain greater concentrations of flavonoids compared with corn-based feeds (Bhagwat and Haytowitz 2015; Harlow et al. 2022). Studies have reported partial or complete reversal of ergot alkaloid-induced vasoconstriction in ruminants supplemented with legumes containing isoflavones, including SBM (Aiken et al. 2016; Harlow et al. 2022; Davis et al. 2023). However, the mode of action of these responses was unclear because of confounding effects on DM and nutrient intake and potential phytoestrogenic properties of isoflavones in the vasculature (Wu et al. 2010; Aiken et al. 2016). In the current study, the total aglycone dose was 0.443 mg/kg of BW in TE + SBH, 3.04 mg/kg of BW in TE + WSB, and 4.42 mg/kg of BW in TE + SBM. These doses were similar to those reported by Harlow et al. (2022) (3.39 mg/kg of BW; SBM) and greater than those reported by Davis et al. (2023) (0.47 mg/kg of BW; red clover). In both studies, supplementation with legumes (eg SBM and red clover) mitigated ergot alkaloid-induced vasoconstriction. It could be possible that partial attenuation of ergot alkaloid-induced vasoconstriction observed in steers receiving TE + SBM was mediated by the antimicrobial and/or vasodilatory properties of isoflavones. However, the mechanisms of vascular relaxation with isoflavones have not been well-studied in ruminants. One study did not observe vascular responses to isoflavones using isolated bovine mesenteric blood vessels (Jia et al. 2015).

### Serotonin metabolism

Increased ruminal tryptophan availability with SBM supplementation could have occurred because of increased dietary supply of tryptophan from SBM and/or inhibition of ruminal amino acid-utilizing bacteria by isoflavones in SBM (Flythe et al. 2013; Harlow et al. 2017). Estimated total tryptophan intake was 3.28 g/d in both NTE and TE, 3.70 g/d in TE + SBH, 7.84 g/d in TE + WSB, and 9.30 g/d in TE + SBM in the current study. Estimates of tryptophan degradation by ruminal microbes are approximately 35% to 50% (Yokoyama and Carlson 1974; Mohammed et al. 2003; Ma et al. 2012); however, ruminal tryptophan degradation is strongly inhibited by gram-negative bactericidal compounds such as monensin (Hammond and Carlson 1980). More recently, Jardon et al. (2025) reported that duodenal tryptophan flow from a high-concentrate diet containing SBM was greater than dietary tryptophan intake in steers. Additionally, 89% to 94% of the net duodenal tryptophan flow from SBM-containing diets was not from microbial origin, suggesting that tryptophan was mostly ruminally undegradable. Therefore, it could be speculated that SBM may have increased postruminal tryptophan flow and subsequent serotonin synthesis in enterochromaffin cells of the small intestine in the current study (Valente et al. 2020), resulting in the observed serum serotonin. The low degradability of tryptophan in the rumen, the presence of monensin in the basal diet, the increased supply of tryptophan from WSB and SBM, and the increased concentrations of isoflavones with SBH, WSB, and SBM support this hypothesis. However, serum tryptophan concentrations were only numerically greater with soybean-containing treatments compared with NTE and TE.

The effects of SBM supplementation on the mitigation of ergot alkaloid-induced vasoconstriction could result from several factors, including direct effects of serotonin on vascular relaxation, direct effects of isoflavones on vascular relaxation, or indirect effects of isoflavones on ruminal tryptophan metabolism. In prior experiments, ergot alkaloid ingestion decreased feed intake and serum or plasma serotonin concentration in cattle (Valente et al. 2020, 2024a). Moreover, increasing exposure to ergot alkaloids increases vasoconstriction of isolated bovine peripheral blood vessels via activation of serotonergic receptors involved in contraction, 5-hydroxytryptamine receptor 2A (HTR2A) (Klotz et al. 2013; Trotta et al. 2018). Increasing serotonin concentration after ergot alkaloid exposure results in dose-dependent vasorelaxation of isolated bovine blood vessels (Trotta et al. 2023), which is mediated via the activation of serotonergic receptors involved in relaxation, 5-hydroxytryptamine receptor 4 (HTR4) (Trotta et al. 2024). Soybean meal supplementation increased serum serotonin concentration compared with TE by 1.42 µM. Previous research demonstrated that a 1 µM increase in extracellular serotonin concentration could elicit a maximal relaxation of isolated bovine peripheral blood vessels via HTR4 activation (Trotta et al. 2024). Overall, these data suggest that the increased vasodilation occurring with SBM supplementation during fescue toxicosis could be partially mediated by increased serotonin.

One possible explanation for the increase in both serotonin and kynurenine with SBM supplementation would be a greater availability of tryptophan to the animal, despite no apparent increase in serum tryptophan concentration. Tryptophan is the sole precursor for serotonin synthesis, which occurs predominantly in the distal gastrointestinal tract (approximately 90%) and to a lesser extent in the central nervous system (about 10%) (Boadle-Biber 1993). The rate-limiting step of serotonin synthesis is the hydroxylation of tryptophan to 5-hydroxytryptophan by tryptophan hydroxylase 1. This intermediate is then decarboxylated by aromatic L-amino acid decarboxylase to form serotonin (Roth et al. 2021). Importantly, serotonin synthesis competes with the kynurenine pathway. In healthy adult mammals, more than 95% of dietary tryptophan is catabolized primarily in the liver through the kynurenine pathway (Yao et al. 2011). Taken together, the increases in serum serotonin and kynurenine concentrations suggest that intestinal serotonin and hepatic kynurenine pathways were stimulated by SBM supplementation, which resulted in increased utilization of tryptophan.

Serum concentrations of 5-HTP and 5-HIAA tended to increase in steers receiving TE compared with NTE. In contrast, previous studies did not observe changes in serum 5-HTP or 5-HIAA concentrations in cattle challenged with TE (Valente et al. 2020, 2024a). 5-HIAA is the primary product of serotonin catabolism, formed by the sequential actions of monoamine oxidase and aldehyde dehydrogenase, which metabolizes serotonin to 5-hydroxyindole acetaldehyde and then to 5-HIAA for urinary excretion (O’Mahony et al. 2015). Increased serum 5-HIAA concentration with TE suggests that ergot alkaloids may have increased serotonin catabolism in the current study. This may be partially responsible for the decreased serotonin observed in fescue toxicosis (Valente et al. 2020). The degradation of serotonin serves as a mechanism to inhibit serotonergic signaling and is regulated by monoamine oxidase activity (Scotton et al. 2019). Considering that exposure to ergot alkaloids causes vasoconstriction in bovine peripheral blood vessels via activation of HTR2A (Klotz et al. 2013; Trotta et al. 2018), we speculate that the increase in serotonin catabolism, evidenced by elevated 5-HIAA, may potentially represent an attempt to attenuate serotonergic activity resulting from persistent stimulation with increased ergot alkaloid interactions (Shi et al. 2008; Valente et al. 2021).

### Feed intake behavior and susceptibility to heat stress

It is well-documented that heat stress and ergot alkaloid ingestion result in a reduction in feed intake (Klotz 2015; Ahn et al. 2020; Valente et al. 2024a, 2024b). To avoid confounding the intake depression induced by heat stress and ergot alkaloids, all steers were exposed to identical environmental conditions and were pair-fed to the lowest DM intake observed from the previous day. Consequently, there were no differences in either basal diet DM intake or total diet DM intake among treatments. This ensures that the differences reported here are attributable to the nutritive value and/or bioactive compounds of soybean feeds, rather than indirect effects resulting from changes in voluntary feed intake.

Despite the absence of differences in feed intake, feeding behavior was influenced by ergot alkaloids and the inclusion of soy-based feeds. Steers receiving TE had a greater number of meals per day that tended to be shorter in duration and smaller in size compared with steers receiving NTE. Previous studies have reported an increase in number of meals (Ahn et al. 2020) and decreased grazing time (Schmidt and Osborn 1993; Seman et al. 1997) in cattle exposed to ergot alkaloids. According to the theory of minimal discomfort in production animals (Forbes 2007), the greater meal frequency observed in TE steers may represent an adaptive response to minimize discomfort associated with ergot alkaloid exposure. Notably, supplementation of SBH restored feeding behavior to a pattern comparable to NTE, whereas SBM supplementation produced an intermediate response. The mechanisms underlying the altered feeding behavior in steers receiving TE, as well as its restoration with soybean supplementation, require further investigation.

One of the key issues associated with ergot-induced vasoconstriction is the reduced ability of animals to thermoregulate, which can exacerbate the negative effects of heat stress (Hemken et al. 1981; Al-Haidary et al. 2001; Ferguson et al. 2021). Several studies have reported decreased skin temperature at peripheral sites in cattle exposed to ergot alkaloids under heat stress conditions (Al-Haidary et al. 1995; Browning et al. 1998; Poudel et al. 2025). In the current study, steers receiving TE exhibited decreased rump skin temperature compared with NTE, whereas TE + SBM supplementation increased rump skin temperature compared with TE steers. The decrease in rump skin temperature observed in TE steers could indicate impaired heat dissipation and may potentially be related to reduced peripheral blood flow. These findings may relate to previous research demonstrating that ergot alkaloid ingestion results in decreased whole-body heat production under fasting (Koontz et al. 2013) and low energy intake conditions (Koontz et al. 2015). Increased rump temperature with TE + SBM suggests that SBM supplementation during fescue toxicosis increased heat dissipation.

### Markers of nutrient metabolism and inflammation

In the present study, steers receiving TE had increased plasma glucose concentrations compared with NTE steers. The effects of ergot alkaloids on plasma glucose concentration in cattle have been variable. Previous studies have reported either unchanged serum glucose (Oliver et al. 2000), increased plasma glucose (Browning 2003; Eisemann et al. 2014; King et al. 2024), or decreased serum glucose (Jackson et al. 2015) in cattle challenged with ergot alkaloids. Such discrepancies may be attributed to differences in DM intake between NTE and TE treatments, the dose of ergot alkaloids, and the duration of exposure. Increased circulating glucose during fescue toxicosis might be attributed to reduced insulin sensitivity and possibly impaired glucose uptake by peripheral tissues (Ferguson et al. 2023; King et al. 2024). Ergot alkaloids from TE increase the hepatic expression of phosphoenolpyruvate carboxykinase, a key regulatory enzyme of gluconeogenesis (Brown et al. 2009). Therefore, increased plasma glucose concentration with TE in the current study might have resulted from greater gluconeogenesis, reduced insulin sensitivity, or a combination of factors. Greater plasma glucose concentration with TE may reflect increased mobilization of energy for use by peripheral tissues.

Additionally, we observed decreased plasma urea concentration in TE steers compared with NTE. Similarly, King et al. (2024) reported decreased plasma NEFA concentration in steers receiving increasing concentrations of ergovaline. Although a direct effect of ergot alkaloids on plasma urea and NEFA concentrations cannot be concluded from the present study, we speculate that the increased number of meals consumed throughout the day by TE steers may have resulted in a more distributed nutrient intake, thereby reducing peaks in urea and NEFA compared with NTE steers, which consumed their feed more rapidly. Among the steers supplemented with soybeans, plasma urea concentration was least in TE + SBH, intermediate in TE + WSB, and greatest in TE + SBM. This reflects a direct effect of increased protein intake due to the greater crude protein concentration of the soybean supplements.

Previous research in cattle has demonstrated that increased heat stress and nutrient restriction increase gastrointestinal permeability (Koch et al. 2019; Fontoura et al. 2022; Bertens et al. 2024). Because of the increased environmental temperature and decreased feed intake with fescue toxicosis in field conditions, it is reasonable to speculate that ergot alkaloids may exacerbate alterations in gut barrier function and intestinal permeability because of increased vasoconstriction of ruminal and mesenteric vasculature in cattle (Trotta et al. 2018; Trotta et al. 2023). Conversely, no differences were observed between TE and NTE groups regarding markers of inflammatory responses and immune system activation. It is likely that the fescue toxicosis challenge of the current study was not successful at eliciting an immune response. This is evidenced by: 1) a lack of change in positive inflammation markers, 2) a lack of change in negative inflammation markers, and 3) a lack of change in total-tract gastrointestinal permeability to Cr-EDTA. Reasons for the lack of response could be too short of a challenge period (7 d) to elicit an immune response or a lack of measurements at baseline to observe changes with the fescue toxicosis challenge. Despite the combined stress of ergot alkaloids, heat stress, and feed restriction, the effects of soybean feeds on decreasing systemic immune activation could not be adequately evaluated in the current study.

## Conclusion

Dietary supplementation of soybean-based feeds has the potential to mitigate fescue toxicosis in cattle. Among the tested feeds (SBH, WSB, SBM), SBM was the most effective. Soybean meal supplementation mitigated ergot alkaloid-induced vasoconstriction and promoted vasodilation in steers challenged with ergot alkaloids from toxic endophyte-infected tall fescue seed. This was evidenced by increased caudal artery cross-sectional area, a tendency for increased blood flow, decreased systolic blood pressure, and increased heart rate with SBM supplementation during fescue toxicosis. Increased serum serotonin concentration may be associated with positive responses observed with SBM supplementation. More research is needed to investigate if the mitigation of ergot alkaloid-induced vasoconstriction with SBM is mediated by an increased dietary supply of essential amino acids, isoflavones, or a combination of both compounds. This will aid in efforts to develop dietary strategies to mitigate fescue toxicosis in beef cattle and utilize widely available feedstuffs for functional inclusion in beef cattle diets.

## Supplementary Material

skag108_Supplementary_Data

## Abbreviations:

5-HIAA: 5-hydroxyindolacetic acid
5-HTP: 5-hydroxytryptophan
AICC: akaike’s information criterion with correction
ATP: adenosine triphosphate
AUC: area under the curve
BW: body weight
Cr-EDTA: chromium-ethylenediaminetetraacetic acid
DM: dry matter
HTR2A: 5-hydroxytryptamine receptor 2A
HTR4: 5-hydroxytryptamine receptor 4
MAO: monoamine oxidase
NEFA: non-esterified fatty acid
NTE: non-toxic endophyte-infected tall fescue
SBH: soybean hulls
SBM: soybean meal
TE: toxic endophyte-infected tall fescue
WSB: whole soybean
