# Supplementary material for: Soybean meal mitigates vasoconstriction and serotonin suppression during fescue toxicosis in beef cattle
Source: J Anim Sci. 2026 Apr 10;104:skag108. doi: 10.1093/jas/skag108 (PMC13188998; doi:10.1093/jas/skag108)
Supplement: skag108_Supplementary_Data [file skag108_supplementary_data.docx]

***Non-esterified fatty acids***

Non-esterified fatty acid concentration was analyzed using an enzymatic method (LabAssay NEFA kit; FUJIFILM Wako Pure Chemical Corporation, Richmond, VA, USA) adapted for use with a multi-mode plate reader (BioTek Synergy HTX; Agilent Technologies Inc., Santa Clara, CA, USA). All reagents and plasma samples were allowed to warm up to room temperature. Color Reagent A was prepared by reconstituting one vial of Chromogen Reagent A (containing acyl-CoA synthetase, coenzyme A, ATP, 4-aminoantipyrine, ascorbate oxidase, and sodium azide) with Solvent A (phosphate buffer containing sodium azide) to a final volume of 10 mL. Reagent B was prepared by reconstituting one vial of Chromogen Reagent B (containing acyl-CoA oxidase and peroxidase) with Solvent B (3-methyl-N-ethyl-N-(β-hydroxyethyl)-aniline) to a final volume of 20 mL. A non-esterified fatty acid standard solution (N294-94351, FUJIFILM Wako Chemicals) was serially diluted to final concentrations of 0.05, 0.10, 0.20, 0.30, 0.40, and 0.50 mM for standard curve construction. For each assay, four microliters of blank (water), standards, or plasma samples was dispensed into the wells of a 96-well microplate, followed by 80 μL of Color Reagent A. Plates were shaken for 60 s, incubated at 37 °C for 10 min, and then ejected from the plate reader. Subsequently, 160 μL of Reagent B was added to each well, followed by another 60 s shaking and a 10 min incubation at 37 °C. Absorbance was then measured at 550 nm. The absorbance of each standard and sample was corrected by subtracting the blank absorbance. Corrected absorbances of standards were linearly regressed against non-esterified fatty acid concentration standards. Plasma non-esterified fatty acid concentrations were calculated from the regression slope.

***β-hydroxybutyrate***

β-hydroxybutyrate concentrations were analyzed using an enzymatic colorimetric method (Fisherbrand Liquicolor β-Hydroxybutyrate, Stanbio Laboratory L.P., Boerne, TX, USA) adapted for use with a multi-mode plate reader (BioTek Synergy HTX; Agilent Technologies Inc., Santa Clara, CA, USA). All reagents and plasma samples were equilibrated to room temperature prior to analysis. A β-hydroxybutyrate standard stock solution (1 mM sodium D-3-hydroxybutyrate) was serially diluted to final concentrations of 0.05, 0.10, 0.20, 0.35, 0.50, and 1.00 mM for standard curve construction. For each assay, five microliters of blank (water), standard, or plasma sample was dispensed into wells of a 96-well microplate, followed by 195 μL of enzyme reagent containing β-hydroxybutyrate dehydrogenase and diaphorase. Plates were shaken for 10 s, incubated at 37 °C for 1 min 20 s, and absorbance was recorded at 505 nm (Read 1). Subsequently, 33 μL of catalyst reagent containing NAD, iodonitrotetrazolium chloride (INT), and oxalate was added to each well. Plates were shaken for 10 s, incubated at 37 °C for 5 min, and absorbance was recorded at 505 nm (Read 2). Absorbance values were corrected by subtracting blank readings, and the change in absorbance (Read 2 – Read 1) of standards was linearly regressed against β-hydroxybutyrate concentrations to generate the standard curve. Plasma β-hydroxybutyrate concentrations were calculated from the regression slope.

***Urea***

Plasma urea was quantified using the method of Jung et al. (1975) as modified by Zawada et al. (2009) with further modifications adapted for use with a multi-mode plate reader (BioTek Synergy HTX; Agilent Technologies Inc., Santa Clara, CA, USA). Reagent A was prepared by dissolving 60 mg of phthaldialdehyde (P1378; Sigma-Aldrich) and 10 mL of 10% sulfuric acid in a 100 mL volumetric flask containing 50 mL of deionized water. The solution was sonicated for 5 min to facilitate phthaldialdehyde dissolution. After cooling, the solution was brought to volume with deionized water and stored at 4 °C. Reagent B was prepared by dissolving 100 mg of primaquine diphosphate (N160393; Sigma-Aldrich), 400 mg of boric acid (A73; Fisher Chemical), and 11 mL of 10% sulfuric acid in a 100 mL volumetric flask containing 50 mL of deionized water. The solution was cooled, adjusted to volume with deionized water, and stored at 4 °C. On the day of the assay, reagents and samples were equilibrated to room temperature. Plasma (500 µL) was deproteinized with an equal volume of 0.6 N perchloric acid, vortexed, and centrifuged at 20,000 × g for 10 min at 4 °C. The supernatant was collected and transferred to 2 mL tubes. A 100 mM urea stock solution (U15-500; Fisher Scientific) was serially diluted to final concentrations of 0.50, 1.00, 2.00, 3.00, 4.00, 5.00, and 6.00 mM for standard curve construction. The working reagent was prepared immediately before use by mixing equal volumes of reagents A and B. Ten microliters of blank (water), standard, or plasma sample were dispensed into the wells of a 96-well microplate, followed by 200 μL of working reagent. Plates were shaken for 5 s, and absorbance was recorded at 430 nm (Read 1). Plates were then incubated at 37 °C for 19 min 20 s, shaken for 5 s, held for 15 s, and read again at 430 nm (Read 2). The change in absorbance (Read 2 – Read 1) of standards was linearly regressed against urea concentrations to generate the standard curve. Plasma urea concentrations were calculated from the regression slope.

***Amyloid A***

Serum amyloid A was analyzed using an ELISA kit (TP807; Tridelta Phase^TM^ range SAA, Tridelta Development Ltd., Maynooth, Co. Kildare, Ireland.) adapted for use with a multi-mode plate reader (BioTek Synergy HTX; Agilent Technologies Inc., Santa Clara, CA, USA). All reagents and serum samples were allowed to warm up to room temperature prior to assay. The 1 × diluent solution was prepared by diluting 25 mL of 10 × diluent buffer into 225 mL of deionized water. The 1 × wash buffer was prepared by diluting 30 mL of 20 × wash buffer into 570 mL of deionized water. Five microliters of sera were diluted into 5 mL of 1 × diluent solution, resulting in a 1:1000 dilution. Dilution was adjusted as needed. The SAA standard was reconstituted in 1 mL of 1× diluent solution and vortexed. Serial dilutions of the SAA standard were prepared in 1× diluent to yield a standard curve with final concentrations of blank (1× diluent only), 18.8, 37.5, 75.0, 150, and 300 ng/mL. Then, 50 µL of anti-SAA/HRP conjugate was dispensed into the wells of a 96-well microplate, followed by 50 µL of blank, standard, or diluted sample. The plate was covered and incubated (37ºC; 1 h) in a hybridization oven (Model H-9360; Hybaid Ltd., Middlesex, United Kingdom). After incubation, plates were aspirated and washed four times with 1 × wash buffer using a microplate washer plate (BioTek 50 TS Microplate Washer, Agilent Technologies, Santa Clara, CA, USA). Next, 100 µL of TMB substrate were added to each well, and the plates were gently mixed and incubated at 25 °C for 15 min in the dark. After incubation, 100 µL of stop solution was added to each well, and the plates were gently mixed. Absorbance was then measured at 450 nm. The absorbance of each standard and sample was corrected by subtracting the blank absorbance. Corrected absorbances of standards were regressed against SAA concentration standards using a four-parameter logistic model using the BioTek Gen5 software (Agilent Technologies Inc., Santa Clara, CA, USA). Serum amyloid A concentrations were calculated from the adjusted model.

***Haptoglobin***

Serum haptoglobin was analyzed using an ELISA kit (HAPT-11; Cow Haptoglobin ELISA Kit; Life Diagnostics, West Chester, PA, USA) adapted for use with a multi-mode plate reader (BioTek Synergy HTX; Agilent Technologies Inc., Santa Clara, CA, USA). All reagents and serum samples were allowed to warm up to room temperature prior to assay. The 1 × diluent solution was prepared by diluting 5 mL of 10 × diluent buffer into 45 mL deionized water. The 1 × wash buffer was prepared by diluting 50 mL of 20 × wash buffer into 950 mL of deionized water. Serum samples were initially diluted 1:40 by adding 5 µL of serum to 195 µL of 1× diluent, followed by a further dilution to 1:1500 by mixing 8 µL of the 1:40 dilution with 292 µL of 1× diluent. Dilutions were adjusted as needed. The haptoglobin standard was reconstituted by adding 573.5 µL of deionized water and vortexed. Serial dilutions of the haptoglobin standard were prepared in 1× diluent to yield a standard curve with final concentrations of blank (1× diluent only), 3.91, 7.81, 15.63, 31.25, 62.5, 125, and 250 ng/mL. For the assay, 100 µL of blank, standard, or diluted samples were added to each well of a 96-well microplate. Plates were incubated at 25 °C for 45 min with constant shaking at 150 rpm. Plates were ejected, aspirated and washed five times with 1 × wash buffer using a microplate washer plate (BioTek 50 TS Microplate Washer, Agilent Technologies, Santa Clara, CA, USA). Next, 100 µL of HRP-conjugate was added to each well, and plates were incubated again at 25 °C for 45 min with constant shaking (150 rpm). Plates were then aspirated and washed five times as described above. Subsequently, 100 µL of TMB substrate was added to each well, followed by incubation at 25 °C for 20 min with constant shaking at 150 rpm. The reaction was stopped by adding 100 µL of stop solution to each well, and plates were gently mixed. Absorbance was immediately measured at 450 nm. The absorbance of each standard and sample was corrected by subtracting the blank absorbance. Corrected absorbances of standards were regressed against the log_10_ of haptoglobin concentration standards using a four-parameter logistic model using the BioTek Gen5 software (Agilent Technologies Inc., Santa Clara, CA, USA). Serum haptoglobin concentrations were calculated from the adjusted model.

***Albumin***

Plasma albumin was quantified using the bromocresol green (BCG) method (Doumas et al., 1971), adapted for use with a multi-mode plate reader (BioTek Synergy HTX; Agilent Technologies Inc., Santa Clara, CA, USA). The BCG reagent was prepared by dissolving 125 mg of bromocresol green (J65225.06, Thermo Fisher Scientific Inc.) in 1 mL of 0.1 M NaOH, followed by the addition of 250 mL of 0.075 M succinic acid buffer (S-0141, Sigma; pH = 4.2) with stirring, and then 2.5 mL of 30% w/v Brij-35 (203724, Sigma-Aldrich). The solution was brought to a final volume of 1 L with deionized water and stored at 4 °C. Albumin standards were prepared by diluting a 5.0 g/dL albumin stock solution (MAK124B, Sigma-Aldrich) in 1× PBS to final concentrations of 0.5, 1.0, 2.0, 3.0, 4.0, and 5.0 g/dL. On day of the assay, reagents and samples were allowed to warm up to room temperature. Five microliters of blank (water), standard, or plasma sample were dispensed into the wells of a 96-well microplate, followed by 200 μL of BCG reagent. Plates were shaken for 5 s, incubated at 25 °C for 5 min, and absorbance was measured at 620 nm. The absorbance of each standard and sample was corrected by subtracting the blank absorbance. Corrected absorbances of standards were regressed against albumin concentration standards using a four-parameter logistic model using the BioTek Gen5 software (Agilent Technologies Inc., Santa Clara, CA, USA). Plasma albumin concentrations were calculated from the adjusted model.

***Cholesterol***

Plasma cholesterol was analyzed using the Infinity Cholesterol Liquid Stable Reagent (Thermo Fisher Scientific Inc., Waltham, MA, USA) adapted for use with a multi-mode plate reader (BioTek Synergy HTX; Agilent Technologies Inc., Santa Clara, CA, USA). A 200 mg/dL aqueous cholesterol stock solution (N1012; Stanbio Laboratory, EKF Diagnostics USA, Boerne, TX, USA) was serially diluted with deionized water to final concentrations of 40, 80, 120, 160, and 200 mg/dL for standard curve construction. On the day of the assay, reagents and samples were allowed to warm up to room temperature. Five microliters of blank (water), standard, or plasma sample were dispensed into wells of a 96-well microplate, followed by 300 μL of Infinity Cholesterol Liquid Stable Reagent (Thermo Fisher Scientific Inc., Waltham, MA, USA). Plates were shaken for 5 s, incubated at 37 °C for 5 min, and absorbance was measured at 500 nm. The absorbance of each standard and sample was corrected by subtracting the blank, and corrected absorbances of standards were linearly regressed against cholesterol concentrations to generate the standard curve. Plasma cholesterol concentrations were calculated from the regression slope.

***Total bilirubin***

Serum total bilirubin was analyzed using the Jendrassik-Grof diazo method (Shull et al., 1980), adapted for use with a multi-mode plate reader (BioTek Synergy HTX; Agilent Technologies Inc., Santa Clara, CA, USA). Reagent A was prepared by dissolving 5 g of sulfanilic acid in 0.1 N HCl to 1000 mL. Reagent B was prepared by dissolving 0.5 g of sodium nitrite in deionized water to 1000 mL. Both solutions were stored at 4 °C in the dark wrapped in aluminum foil. The accelerator solution was prepared by dissolving 50 g of caffeine and 75 g of sodium benzoate in 0.1 M acetate buffer (pH = 4.7) under heating with stirring. The solution was brought to 1000 mL and stored under the same conditions. A 10 mg/dL bilirubin stock solution was prepared by dissolving 2.58 mg of bilirubin (A17522.03; Thermo Fisher Scientific, Ward Hill, MA, USA) in a 25 mL volumetric flask containing 0.1 NaOH + 1% BSA. The stock was brought to volume and stored at -20 °C in the dark. On day of the essay, reagents and samples were allowed to warm up to room temperature. Stock bilirubin solution was serially diluted with 1× PBS to 0.5, 1.0, 1.5, 2.0, 3.0, and 4.0 mg/dL The working diazo reagent was freshly prepared by mixing 10 mL of reagent A with 0.3 mL of reagent B. Ten microliters of blank (1 × PBS), standard, or serum sample were dispensed into the wells of a 96-well microplate, followed by 50 μL of accelerator reagent. Then, 50 μL of diazo reagent was added to standards and samples wells, while 50 μL of 0.1 N HCl were added to blank wells. Plates were shaken for 10 s, incubated at 25ºC for 10 min in the dark, and the absorbance was recorded at 560 nm. The absorbance of each standard and sample was corrected by subtracting the blank, and corrected absorbances of standards were linearly regressed against total bilirubin concentrations to generate the standard curve. Total serum bilirubin concentrations were calculated from the regression slope.
